# Interest of URS-L in the Treatment of Ureterolithiasis in Preschool Children

**DOI:** 10.3389/fped.2019.00324

**Published:** 2019-09-06

**Authors:** Adam Halinski, Andrzej Halinski, Marcin Zaniew, Bartosz Kudliński, Jolanta Soltysiak, Bartłomiej Sobolewski, Henri Steyaert

**Affiliations:** ^1^Department of Paediatric Urology, “Klinika Wisniowa”, “Cherry Clinic”, Zielona Gora, Poland; ^2^Clinical Department of Paediatric Surgery and Urology, University Hospital in Zielona Góra, Zielona Góra, Poland; ^3^Department of Paediatrics, University of Zielona Góra, Zielona Góra, Poland; ^4^Department of Pediatric Nephrology and Hypertension, Poznan University of Medical Sciences, Poznan, Poland; ^5^Department of Pediatric Surgery, Queen Fabiola Children's University Hospital, Université Libre de Bruxelles (ULB), Brussels, Belgium

**Keywords:** stone disease, laser, ureterorenoscopy, preschool children, DJ catheter

## Abstract

Urolithiasis can affect all children even preschool ones. Diagnostic difficulties in the youngest children are due to the problems in locating pain and determining its character and severity. In keeping with the ALARA (As Low As Reasonably Achievable) protocol, the number of imaging tests possible to perform is very limited. Ultrasound is the first line exam of choice. After diagnosis of the presence of a stone, ESWL (Extracorporeal Shock Wave Lithotrypsy) should always be considered and offered to parents due to its high effectiveness and minimal invasiveness. If ESWL is contraindicated or not well-accepted by parents, authors suggest another minimal invasive approach: URS-L (Uretherorenoscopy–Lithotrypsy). Our study clinically analyzes 87 children, which were treated between 2009 and 2017 using the URS-L procedure. URS-L treatments were performed using Lithoclast until 2009, and after that time, using the holmium laser Ho:YAG. The overall effectiveness of treatments was 93.3%. There was no failure in the access to the stones. A macroscopic hematuria (Clavien-Dindo I grade) was observed through the second post-operative day in 9.2% of treated patients. No urosepsis was observed. Full metabolic evaluation was performed on all patients. Children remained under constant urological and nephrological observation. A recurrence of urolithiasis was observed in 35.6% of the cases. Treating ureteral lithiasis in young infants remains a big challenge. Our series shows that modern minimal invasive techniques used by very experienced pediatric urologists in high volume centers gives excellent results. In most cases, surgery should no longer need to be an option.

## Introduction

In recent years, a number of international clinical researches and available publications have shown a steady increase in the number of urolithiasis in children in all age groups (1). This trend warrants diagnostic vigilance in all cases of abdominal pain. A unique clinical situation is observed in small patients as they are unable to locate their pain and cannot adequately communicate its character or severity. Most described symptoms are crying, anxiety, vomiting, hematuria, traces of blood or small stones on the diaper or recurrent urinary tract infection (UTI) with painful micturition. Available tests for adults, i.e., Uro-CT (Computed Tomography with a urography phase), low dose NCCT (No Contrast Computed Tomography), or IVU (urography), are not always applicable to children, especially because they cause irradiation (Table 1). Treatment options are numerous, going from ESWL (Extracorporeal Shock Wave Lithotrypsy) to open surgery. The aim of this study is to present a clinical analysis, demonstrating difficulties and limitations, both in the diagnosis and treatment, of ureterolithiasis in children from 0 to 6 years of age (preschool children) and to propose a new minimal invasive treatment able to replace surgery in most cases.

**Table 1:** Mean radiation doses of different radiological exams (EAU Guidelines 2018).

**Method**	**Radiation exposure (mSv)**
KUB (kidney-ureter-bladder) radiography	0.5–1
IVU	1.3–3.5
Tomography with contrast	25–35
Tomography without contrast—regular dose CT	4.5–5
Tomography without contrast low dose—NCCT	0.97–1.9

## Materials and Methods

**Figure 1:**
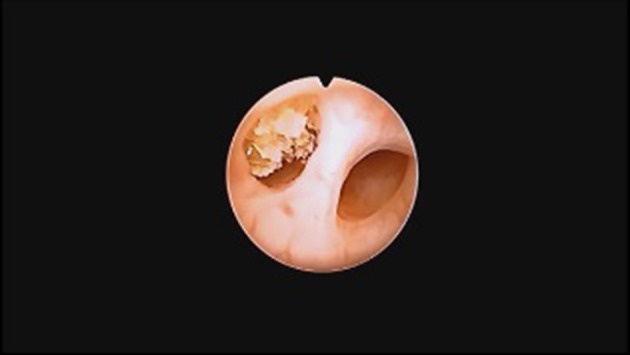
Stone in a bifid ureter in 4 year's old boy.

This is a retrospective study. It includes 87 children treated between 2009 and 2017 in the Clinical Department of Pediatric Surgery and Urology, University Hospital in Zielona Góra, where a semi-rigid ureterorenoscopy (URS-L) was used to remove stones (Figure 1). Chart review was performed to collect data on symptoms, imaging, location of the stones, laboratory tests, type of treatment, complications, and recurrences. URS-L Technique: Every patient underwent general anesthesia using a laryngeal mask. Every patient was administered a balanced crystalloid fluid at a dose of 4–5 ml/kg. Intraoperative monitoring consisted in: pulse oximetry, EKG, capnography, non-invasive blood pressure measurement, and concentration of anesthetic gases. Post-operative analgesia was provided with intravenous infusion of acetaminophen at a dose of 15 mg/kg. Stone management was performed using two ureterorenoscopes: 6F, with two working channels and 4.5 F (2). A fluoroscopy device was always placed over the operating table, ready to be used in case of need. Urethrocystoscopy was always performed as the first step. We used routine hydrophilic safety wires, considering that this is a mandatory part of the technique for the URS-L procedure, especially in small children. We used only gravity irrigation. We did not use any equipment for forced irrigation. Rarely, small flushes with 2 ml (more common) or 5 ml syringe were provided. After 2009, only a Ho:YAG laser with a 272 nm fiber was used for stone disintegration. A Foley catheter was inserted at the end of surgery for <24 h in all cases.

## Results

Children were referred from various centers. The mean stone size was 8.8 mm (range 4–15 mm). We performed a semi-rigid ureterorenoscopy procedure in 87 children, of whom 18 underwent prior ESWL (20.6%). All procedures were conducted under general anesthesia. None of the patients required endotracheal intubation or the use of striated muscle-relaxing drugs. Thirty percent of patients needed an intravenous bolus injection of propofol, at a dose of 20–30 mg. There were no episodes of cardiac arhythmia observed on electrocardiogram in any of the patients during the procedure, and for 60 min thereafter. The youngest patient in this group was 5 months old, and the oldest was 72 months (mean: 28.2 months). Lithiasis occurred in the left ureter in 46 cases, and in the right ureter for 41 cases. Hydronephrosis was observed in 78 patients, i.e., 89.6% (Table 2). All patients underwent an abdominal diagnostic ultrasound scan. Urography was performed in 2 patients, the last one being in 2009. Low dose NCCT was performed in 7 patients. In general, blood samples were within normal limits. Urine tests frequently showed an increase in erythrocytes.

**Table 2:** Stone location and effectiveness of the endoscopic procedure.

	**Upper ureter**	**Middle ureter**	**Lower ureter**	
Balistic device	2	4	9	15
Effective treatment	1	3	9	13
%	50%	75%	100%	86.67%
Laser device	8	14	50	72
Effective treatment	5	12	49	66
%	62.5%	85.70%	98%	91.67%

The duration of the procedure ranged from 15 to 55 min (mean 29.6 min). The ureter was freed in all patients. In 77 children (i.e., 88.5%), a DJ (double J) catheter was implemented, which was then removed within 14 days post-operatively. No colic pain was observed in this group. In 10 children no DJ catheter was left in place. Of these 10 children, colic pain was observed in eight. Two of all patients suffered from post-operative vomiting within 60 min after the end of the procedure. No anti-emetic prevention protocol was implemented. There was a statistically significant difference in analgesic intake after surgery in the group of children with the DJ catheter vs. without the DJ catheter (*p* < 0.05). No serious complications were observed during the intraoperative and post-operative periods in both groups.

Given the two types of lithotripsy devices used, we divided patients into two groups. The first group was designated the ballistic device group, while the second group was designated the laser group. Fifteen URS-L procedures were performed using ballistic device and 72 procedures used the holmium laser (after acquisition of such a device). Full disintegration of deposits occurred in 13 patients from the first group. One partial disintegration was observed with displacement of the rest of the deposit up to the kidney. One procedure was performed as a “push up” surgery and the stone was disintegrated using ESWL as the next step. In the second group full disintegration of the deposit (with the use of holmium laser) in the ureter was achieved in 66 children. In five children partial disintegration was observed with the displacement of the rest of the deposit up with to the kidney using the “push up” method. The effectiveness of the URS-L surgery in the first group was therefore 86.67%, while it was 91.67% in the second group (laser). Surgery with laser was significantly shorter: ballistic device times ranged from 20 to 55 min (mean 31.4 min) vs. 15–22 min (mean 16.2 min) for the laser group. After “push up,” URS-L stones were disintegrated using the ESWL method. In all “push up” procedures DJ stent was left in the ureter. In all children, the ureters were stone free (100%). Fever was observed in two cases after the procedure (2.2%). Transient hematuria up to the second day was observed in 8 children (9.2%). During ultrasonography at follow-up, no urine retention was observed in the calico-pelvic system. Length of hospital stay ranged from 2 to 4 days (mean 2.2 days). All removed deposits were evaluated for their chemical composition. All children were referred for a nephrological analysis after surgery. Patients underwent constant surveillance of the Pediatric Nephrology and Pediatric Urology Clinic. The shortest follow up was 1 year and the longest was 9 years and 5 months, with a mean follow up of 3.5 years. Recurrence of urolithiasis was observed in 35.6% of our cases during follow up.

## Discussion

The wide range of symptoms and the unability to really indicate the location of the pain, the diagnosis of ureteral stones is a very difficult task for physicians seeing patients in this age group. Laboratory findings, especially in infants, are mostly within normal limits. Urine tests are more often positive, showing at least some increase in the erythrocyte count. Through ultrasound examination, we can observe hydronephrosis, and in some favorable conditions, location of an obstacle in the outflow of urine in the form of a stone is discovered. However, ultrasound is unfortunately not conclusive in several cases. CT scan without anesthesia is difficult in this age group due to the need for temporary immobility. It also involves a radiation dose, and the test itself has limited sensitivity and specificity. In some cases, the child suffers from colic pain without stagnation of any urine and without any stone shadow. When such cases are suspected, low dose NCCT can be a good option to detect the presence of a stone. The radiation dose is lower than for an urography, while the sensitivity and specificity is close to 100% for deposits in the ureter >3 mm (3). However, an experienced radiologist is needed for a good interpretation of the low dose CT scan and measurement of the Hounsfield units (HU) of the stones (4).

According to the EAU (European Association of Urology) guidelines, ultrasound is the test of choice, which should include the kidneys, a well-filled bladder, and sections of the ureters (especially proximal and distal). If the ultrasound examination is not conclusive, further imaging tests should be considered. Their use and purposefulness should be weighed individually depending on the availability at the hospital, and the experience of the radiologist. Irradiation is a concern due to the high risk of recurrence of lithiasis during childhood. This is why authors used the ALARA protocol (As Low as Reasonably Achievable) is used, which aims to limit X-ray tests (5).

In children, the treatment of choice, both for proximal ureteral stones and renal calculi is ESWL under general anesthesia (6). Unfortunately, locating the stone during the procedure sometimes turns out to be difficult or the stone does not respond to the extracorporeal treatment. In our algorithm, after parent consent, we perform one ESWL procedure if the stone is above the junction with the iliac vessels and is clearly visible during ultrasound examination. If we do not observe a response to the extracorporeal wave procedure, if the stone is located in the lower part of the ureter or if urolithiasis is not clearly visible using sonography, we offer to parents the option of URS-L surgery. Particularly when the stone is located in the distal ureter, we believe the URS-L procedure should always be recommended as the treatment of choice (7). In such cases, especially in girls, ESWL should be contraindicated. In the case of a patient with suspected urinary tract infection or sepsis, urgent urinary diversion should be performed through a nephrostomy or a DJ catheter, and endoscopic procedure should be delayed (8).

Effectiveness of ESWL compared to URS-L are reported around 70–80% vs. 86–100%, respectively (9). In our center URS-L was effective in 90% of the cases. Use of Holmium laser increased the success rate in comparison with the clast technique and made the intervention shorter. Results of our totally minimal invasive approach for stone treatment are comparable with the literature (10, 11). However, few publications studying this age group exist (9). During follow-up, we did not observe any complications, such as ureteral stricture formation or VUR appearance (12). Of course, long-term follow up and prospective studies are mandatory to confirm these results. Another interesting point must be noticed. Children who had a JJ stent post-operatively suffered less from colic pain than those who had no stenting.

Metabolic evaluation after surgery is very important because of the high rates in children presenting with urinary stones. A urinary stone might be the first manifestation of numerous pathologies and metabolic disorders. Metabolic abnormalities that increase the risk of nephrolithiasis can be identified in 75–84% of children with such a presentation. The most commonly reported of such diseases are hypercalciuria and hypocitraturia (4, 13). Nonetheless, low urine volume is another common and very important finding in urine analysis and should not be forgotten. The role of diet is inherently connected to metabolic abnormalities. Drinking less water and greater daily intake of sodium are the well-known risk factors for kidney stones (14). Excessive animal protein and fructose intake, as well as increasing obesity in adolescents, are also contributing factors (4, 15). Other causes of nephrolithiasis such as taking calcitriol, steroids, antiretrovirals, vitamin C and D, and even furosemide should always be considered. Inflammatory bowel disease, cystic fibrosis, renal dysplasia are also associated with stone formation.

For this reason, the American Urological Association (AUA) guidelines recommend a screening evaluation consisting of a detailed medical and dietary history, serum chemistries and urinalysis after the first stone event (16). However, in children there are no clearly stated or published guidelines for pediatric stone-formers.

Unfortunately, in some cases, such metabolic investigation of the urine alone may not ensure the actual diagnosis of the lithogenic disease (17). In this situation stone analysis is also essential to allow unambiguous diagnosis (18, 19). For this reason, the AUA recommends a stone analysis at least at once, if a stone is available (16). Stone analysis has to report qualitative and quantitative information regarding crystalline phases, their location within the stone and structural characteristics (17).

Stone disease is an accumulation of environmental exposure, genetic and metabolic predispositions with high risk of recurrence. Pediatric urologists should closely cooperate with nephrologists to perform appropriate metabolic assessment and stone analysis in pediatric stone-formers. This complete evaluation and appropriate diagnosis is necessary to prevent the next episodes of nephrolithiasis in this young age; in particular because the high grade of recurrence of stone disease in young children as demonstrated in our study (>35%).

## Conclusions

Miniaturization and overall progress in endourological equipment (endoscopes, safety wires, DJ stents, and visualization cameras) allows for treatment of even the smallest patients (20). Of course, the surgeon's experience in these procedures cannot be underestimated (21, 22). After literature review, we agree that the diagnosis and treatment of ureterolithiasis in children under the age of 6 may be challenging. Nonetheless, we demonstrate the effectiveness of our minimal invasive protocol. Through analysis of complications, it can be concluded that semi-rigid ureterorenoscopy is a safe and effective tool, even in infants (23). Due to the technical difficulties of the procedure, it should be performed in centers with a large number of patients.

## Data Availability

All datasets generated for this study are included in the manuscript and/or the Supplementary Files.

## Ethics Statement

This retrospective study is in accordance with the ethical standards of the institutional and/or national research committee and with the 1964 Helsinki Declaration and its later amendments or comparable ethical standards.

## Author Contributions

All authors listed have made a substantial, direct and intellectual contribution to the work, and approved it for publication.

### Conflict of Interest Statement

The authors declare that the research was conducted in the absence of any commercial or financial relationships that could be construed as a potential conflict of interest.
